# Vascular access cannulation in hemodialysis patients: technical approach

**DOI:** 10.1590/2175-8239-JBN-2019-0031

**Published:** 2019-12-09

**Authors:** Manuel Carlos Martins Castro, Francisca Tokiko Yanagida Carlquist, Celina de Fátima Silva, Magdaleni Xagoraris, Jerônimo Ruiz Centeno, José Adilson Camargo de Souza

**Affiliations:** 1 Instituto de Nefrologia, Taubaté, São Paulo, SP, Brasil.; 2 Instituto de Nefrologia, São José dos Campos, São Paulo, SP, Brasil.

**Keywords:** Vascular Access Devices, Catheterization, Arteriovenous Fistula, Blood Vessel Prosthesis, Renal Dialysis, Dispositivos de Acesso Vascular, Cateterismo, Fístula Arteriovenosa, Prótese Vascular, Diálise Renal

## Abstract

**Introduction:**

The vascular access cannulation technique varies among clinics, and guidelines on vascular access give little importance to cannulation techniques. The objective of this study was to evaluate the cannulation technique and to determine which factors are associated with each detail of the technique.

**Material and methods:**

The vascular access cannulation was evaluated in 260 patients undergoing hemodialysis. The type and anatomical location of the vascular access, the cannulation technique, direction, gauge, and distance between needles, besides bevel direction and needle rotation were registered.

**Results:**

The arteriovenous fistula was the most frequent vascular access (88%), the most used cannulation technique was area (100%), the needle direction was anterograde in most cases (79.5%), and the mean distance between the tips of needles was 7.57±4.43 cm. For arteriovenous grafts, the proximal anatomical location (brachial artery) and cannulation with 16G needles in anterograde position were more predominant. For arteriovenous fistulas, the distal anatomical location (radial artery) and cannulation through 15G needles were more common. Cannulation of vascular access in retrograde direction was associated with a greater distance between needles (13.2 ± 4.4 vs 6.1 ± 3 cm, *p* < 0.001). Kt/V was higher when the distance between needles was higher than 5 cm (1.61 ± 0.3 vs. 1.47 ± 0.28, *p* < 0.01).

**Conclusions:**

The vascular access cannulation technique depends on the vascular access characteristics and expertise of cannulators. Clinical trials are required for the formulation of guidelines for vascular access cannulation.

## RESUMO

**Introdução:**

A técnica de canulação do acesso vascular varia entre instituições de saúde, e as diretrizes sobre o acesso vascular dão pouca importância às técnicas de canulação. O objetivo deste estudo foi avaliar a técnica de canulação e determinar quais fatores estão associados a cada detalhe da técnica.

**Material e métodos:**

A canulação do acesso vascular foi avaliada em 260 pacientes em hemodiálise. Foram registrados o tipo e localização anatômica do acesso vascular, a técnica de canulação, a direção, a bitola e a distância entre as agulhas, além da direção do bisel e da rotação da agulha.

**Resultados:**

A fístula arteriovenosa foi o acesso vascular mais frequente (88%), a técnica de canulação mais utilizada foi a área (100%), a direção da agulha foi anterógrada na maioria dos casos (79,5%) e a distância média entre as pontas das agulhas foi de 7,57 ± 4,43 cm. Para enxertos arteriovenosos, a localização anatômica proximal (artéria braquial) e a canulação com agulhas 16G em posição anterógrada foram mais predominantes. Para as fístulas arteriovenosas, a localização anatômica distal (artéria radial) e a canulação através de agulhas 15G foram mais comuns. A canulação do acesso vascular na direção retrógrada foi associada a uma maior distância entre as agulhas (13,2 ± 4,4 v.s. 6,1 ± 3 cm, *p* < 0,001). O Kt / V foi maior quando a distância entre as agulhas foi superior a 5 cm (1,61 ± 0,3 vs. 1,47 ± 0,28, *p* < 0,01).

**Conclusões:**

A técnica de canulação do acesso vascular depende das características do acesso vascular e da experiência dos “canuladores”. Ensaios clínicos são necessários para a formulação de diretrizes para a canulação do acesso vascular.

## INTRODUCTION

Arteriovenous fistulas (AVF) and arteriovenous grafts (AVG) are the preferred vascular accesses for patients with chronic kidney disease undergoing hemodialysis. Evidence points to the superiority of AVF and AVG over catheters and, to a lesser extent, for AVF over AVG.^1^^-^ 4

In 2014, the data from the Brazilian Dialysis Census showed that there were 44,616 patients undergoing hemodialysis in 312 dialysis units in Brazil. The AVF was the vascular access type in 35,380 (79.3%) patients and AVG in 1,829 (4.1%).^5^

Many factors influence the survival of the vascular access in hemodialysis. Characteristics of the patient, type of dialytic therapy, and surgical technique, besides the cannulation procedure are involved in the longevity of the vascular access.^6^ Although some studies have shown the importance of the puncture technique in vascular access survival, there is a lack of evidence to support a particular type of cannulation.^7^^,^ 8

Recommendations for vascular access cannulation have received little attention. Aspects such as anatomic location, direction of needle insertion, needle gauge, needle rotation, needle with back eye or not, bevel orientation, and the differences among the three methods for cannulation- rope ladder, area, and buttonhole - are little discussed in the medical literature. This lack of knowledge can be associated with puncture failures and consequent increase in dialysis morbidity and mortality.

In this study, we report our experience in performing vascular access cannulation for hemodialysis and investigate the most important characteristics that influence the cannulation technique.

## MATERIALS AND METHODS

In this cross-sectional study, we analyzed 260 patients on maintenance hemodialysis at the Institute of Nephrology (Inefro) of Taubaté and São José dos Campos, Brazil. The two dialysis units are under the same technical coordination and the nursing staff receive the same training.

The type of vascular access (AVF or AVG), the location of the arteriovenous anastomosis (proximal-brachial artery or distal-radial artery), the direction of the puncture cannula (anterograde or retrograde), the bevel position (up or down), the needles gauge (17G, 16G or 15G), the distance between the tip of needles inside the vascular lumen and, finally, needle rotation or not after cannulation were recorded for each patient. In one patient, the direction of the puncture cannula was not registered and the information was not included. The dialysis dose was assessed by urea Kt/V through the single pool model, using the 2nd generation Daugirdas equation.^9^ The dialysis dose was measured in the same month that the cannulation technique was evaluated.

All patients underwent three hemodialysis sessions per week. The duration of the session was 3.5 to 4 hours. The blood flow rate was 300 to 450 mL/min. The dialysate flow rate was 500 mL/min. The dialysis membranes were of high flux polysulfone with surface area of 1.8 to 2.2 m^2^.

The Kt/V target was ≥ 1.4. To reach this value, adjustments in dialysis prescription were performed in the following sequence: surface area of the filter, blood flow rate, and treatment time. If the minimal dialysis dose was not reached after these measurements, the puncture sites were reviewed to increase the between-needle distance. In this condition, the goal was to increase the distance between puncture sites or perform the cannulation with the needles in retrograde position.

Statistical analyze was performed by Fisher’s exact test to compare categorical variables and by unpaired t-test for numerical variables. The statistical program was GraphPad Prism^(®)^ version 5.0. The data are reported as means±SD for continuous variables and percentages for categorical variables. The level of significance was set at *p* < 0.05.

## RESULTS

Of the 260 patients evaluated in this study, 164 (63.1%) were males and 96 (36.9%) were females. The mean age was 58.9 ± 15.1 years, ranging from 15 to 94 years, and dialysis vintage was 81.7 ± 54.8 months, ranging from 17.2 to 267.4 months.

Table 1 shows the type of vascular access, anatomical location, puncture direction, needles gauge, distance between the tip of needles, and Kt/V value. In our patients, 88.1% had an AVF, in 79.5% the punctures were anterograde, and the mean distance between needles was 7.57 ± 4.43 cm. The anatomical location of the vascular access was distal (radial artery) in 53.5% of patients and proximal (brachial artery) in 46.5%. There was predominance in the use of 15G needles (59.6%) compared to 16G needles (40%). The Kt/V value was 1.58 ± 0.36.

**Table 1:** Characteristics of arteriovenous fistula, arteriovenous graft, and cannulation technique

Number of patients	260
Type	
Fistula	88.1%
Graft	11.9%
Location	
Distal	53.5%
Proximal	46.5%
Needle direction*	
Anterograde	79.5%
Retrograde	20.5%
Needle gauge	
15G	59.6%
16G	40.0%
17G	0.4%
Distance between the tip of needles	7.57 ± 4.43 cm
Kt/V	1.58 ± 0.36

* n=259.

Tables 2 through 6 show the analysis of a particular characteristic of the cannulation technique in relation to the other variables. In AVG the localization was predominantly proximal, the puncture was anterograde, and the needles gauge was 16G (Table 2). The distal vascular accesses were predominantly AVF, punctured with 15G needles, and the distance between needles was larger. In proximal vascular accesses, the anterograde cannulation was predominant (Table 3).

**Table 2:** Characteristics of cannulation technique and dialysis dose in arteriovenous fistulas and arteriovenous graft

Variable	AV Fistula	Graft	*p* value
Kt/V	1.58 ± 0,37	1.57 ± 0,23	NS
Distance of needle (cm)	7.7 ± 4.6	6.2 ± 1.6	NS
Location (proximal/distal)	91/138	30/1	< 0.001
Direction (anterograde/retrograde)	175/53	31/0	< 0.001
Needle gauge (15G/16G)	155/74	0/31	< 0.001

**Table 3:** Characteristics of the cannulation technique and dialysis dose according to anatomical location of the arteriovenous fistula and graft

Variable	Location Distal	Proximal	*p* value
Kt/V	1.56 ± 0.39	1.61 ± 0.32	NS
Distance of needle (cm)	8.7 ± 5.1	6.3 ± 3.1	< 0.001
Type (fistula/graft)	138/1	91/30	< 0.001
Direction (anterograde/retrograde)	99/40	107/13	< 0.001
Needle gauge (15G/16G)	101/38	54/67	< 0.001

**Table 4:** Characteristics of the cannulation technique and dialysis dose according to needle direction

Variable	Anterograde	Retrograde	*p* value
Kt/V	1.57 ± 0.36	1.62 ± 0.36	NS
Distance of needle (cm)	6.1 ± 3.0	13.2 ± 4.4	< 0.001
Type (fistula/graft)	175/31	53/0	< 0.001
Location (proximal/distal)	107/99	13/40	< 0.001
Needle gauge (15G/16G)	112/94	43/11	< 0.001

**Table 5:** Characteristics of cannulation technique and dialysis dose according to needle gauge

Variable	Needle gauge		*p* value
	15G	16G	
Kt/V	1.61 ± 0.39	1.53 ± 0.29	NS
Distance of needle (cm)	8.4 ± 4.7	6.4 ± 3.7	< 0.001
Type (fistula/graft)	155/0	74/31	< 0.001
Location (proximal/distal)	54/101	67/38	< 0.001
Direction (anterograde/retrograde)	112/42	94/11	< 0.001

**Table 6:** Characteristics of the cannulation technique and dialysis dose according to distance between the tip of needles

Variable	Distance of needle (cm)		*p* value
	< 5	≥ 5	
Kt/V	1.47 ± 0.28	1.61 ± 0.30	< 0.01
Type (fistula/graft)	54/4	177/25	NS
Location (proximal/distal)	32/26	90/112	NS
Direction (anterograde/retrograde)	57/1	150/51	< 0.001
Needle gauge (15G/16G)	28/30	127/75	< 0.05

Regarding the direction of the cannula, retrograde puncture with 15G needles predominated in distal AVFs. These characteristics were associated with greater between-needle distance (Table 4). Regarding the caliber, the 15G needle was used in distal AVFs and was associated the greater between-needle distance. In anterograde punctures, 16G needles were more used (Table 5).

Finally, in punctures where the distance between needles was less than 5 cm, the dialysis dose was lower and the anterograde puncture was more used. On the other hand, when the distance was greater than 5 cm, the punctures were performed with 15G cannulas (Table 6).

## DISCUSSION

The main complications associated with vascular access cannulation for HD are thrombosis, hemorrhage, infection, and aneurysmatic dilatation. The experience of the cannulator and technique selected can reduce the frequency of these complications.

Several factors have been associated with survival of the vascular access. 10 ^,^ 11 Younger age, male, non-diabetic patients, with lower body mass index, capable of performing vascular access compression after hemodialysis, without congestive heart failure and without prescription of anticoagulants are factors associated with greater vascular access survival. On the other hand, advanced age, short maturation time, grafts, distal fistula, cannulated fistula with small diameter needles, fistulas that do not allow adequate blood flow, fistulas with greater pressure for venous return, and those with retrograde cannulation are associated with a higher failure rate. Therefore, in addition to the characteristics of the patient, several factors associated with the cannulation technique have an impact on vascular access survival.

Preferably, the vascular access should be constructed using a distal vein in the non-dominant upper limb. However, variations of this rule are very frequent due to increased dialysis survival, previous implantation of central venous catheters, exhaustion of native veins, and increased use of grafts. Depending on the type of vascular access, location, degree of maturation, and extent available for cannulation, the best puncture technique is determined. In addition, cannulation techniques vary among clinics primarily because of training approaches.

Regarding the introduction of the needles, there are three different methods for vascular access cannulation: rope ladder, area, and buttonhole.^12^^-^ 14

The rope ladder technique is the most indicated because it uses the entire extension of the vessel, and is associated with less thrombosis, stenosis, infections, and aneurysmatic dilatations. However, the technique needs an extensive surface for the introduction of the needles. Area puncture involves the introduction of needles into a restricted area. The progressive destruction of nerve fibers in the puncture area results in decreased pain during cannulation; however, it is associated with a higher frequency of aneurysms, pseudoaneurysms, stenosis, thrombosis, and infection, particularly in AVG.^7^

The buttonhole puncture is associated with less pain and may be the method of choice in AVF with reduced extension for puncture and for patients on home dialysis. The technique is associated with a higher infection rate and should not be used for AVG cannulation, as it causes exhaustion of the material and formation of pseudoaneurysms.^15^

In the rope ladder technique, two new sites are chosen for needle insertion in each hemodialysis session. The needles should be at least 5 to 7 cm apart and 4 cm from the arteriovenous anastomosis. There are situations where it is not possible to follow these rules, especially when the length of the vascular access is limited or the vein becomes very deep along its route. Robbin et al.^16^ and Van Loon et al.^10^ suggest that the vein length for puncture should be of at least 10 cm to ensure the rotation and the distance between needles, resulting in greater success rate with minimal complications. In our study, we did not accurately evaluate the length of the puncture surface.

There is no definition of the minimum distance from the previous puncture to consider the cannulation as not being area. This lack of criteria can cause confusion. In fact, dialysis units that believe to use the rope ladder technique may be using area cannulation. Dialysis units with a high prevalence of vascular access aneurysms are probably using area cannulation. We believe that when the needle insertion distance is less than 2.0 cm from the previous cannulation, the technique should be considered area puncture.

The venous needle should be inserted in the direction of blood flow. The arterial needle can point in the two directions. In anterograde cannulation, the arterial needle points to direction of blood flow and in retrograde cannulation the needle points to the arteriovenous anastomosis.

The order of needle insertion depends on expertise and experience of the cannulator. Whenever possible, the venous needle is introduced first to secure the return of the blood to the patient. Arterial cannulation is performed in the region closest to the venous anastomosis and is more susceptible to errors, especially when the introduction of the needle is retrograde. In fistulas with very short cannulation length, the arterial puncture must be performed before the venous one.

Several results of our study are in agreement with those reported by others. Gauly et al^17^, in 2011, published an extensive multicenter study involving 10,807 patients from 171 Fresenius Medical Care clinics, in nine European countries and South Africa. Table 7 shows the main features of cannulation techniques observed in that study compared to our results. Although the fistulas have similar characteristics in relation to type and location, there were differences in the puncture technique that can be explained by local practices and preferences. For example, in our unit, area cannulation is used in 100% of patients. This practice must be modified, since rope ladder cannulation seems to be the ideal technique.

**Table 7:** Cannulation technique of arteriovenous fistula and graft in the Institute of Nephrology (Inefro) compared to literature data

Variable		Inefro (%)	Gauly et al.^17^ (%)
Type of vascular access	Fistula	88.1	91
	Graft	11.9	9
Location of arteriovenous anastomosis	Distal	53.5	49.7
	Proximal	46.5	48.5
Needle gauge	14G	0	2.4
	15G	59.6	61.3
	16G	40	33.2
	17G	0.4	1.7
Cannulation technique	Area	100	61
	Rope ladder	0	31
	Buttonhole	0	6.1
Needle direction	Anterograde	79.5	63
	Retrograde	20.5	37
Bevel	Up	100	72.3
	Down	0	27
Needle rotation	Yes	100 (arterial)	43.2
	No	100 (venous)	54.5
Back eye	Yes	100	65
	No	0	30.3
Distance between needles (cm)		7.6 ± 4.4*	7.0 ± 3.7

* Distance between the tip of the needles inside the fistula.

AVGs are reserved for patients with distal vein deprivation. In addition, the graft requires a high blood flow rate to reduce the risk of intravascular thrombosis; consequently, most of them are constructed using the brachial artery.^18^^,^ 19 In our center, 97% of the grafts are proximal, and cannulated with the anterograde technique (100%), using 16G needles (100%). This puncture technique is probably associated with fear of trauma during cannulation, development of pseudoaneurysms, and high risk of bleeding after needle withdrawal.

In our center, the distal vascular access is mainly an AVF (99%) and the distance between needles is greater. This is probably associated with the greater extension of the puncture surface, and cannulation with retrograde direction of the needles (40%). In proximal fistulas, the between-needle distance was lower because they are AVG (25%), possibly with a smaller surface for cannulation, and punctured with needles in anterograde position (89%).

In our study, anterograde puncture was associated with shorter distance between needles’ tip, probably because native (85%) and proximal (52%) fistulas predominated, a condition where it is technically easier to perform an anterograde cannulation.

In our center, 15G gauge needles are used in AVFs (100%), distal cannulations (65%), and cannulations with needles in anterograde position (73%), probably because the retrograde puncture is subject to a higher frequency of serious complications when the caliber of the needle is larger.

The main determinants of hemodialysis dose (Kt/V) are treatment time, filter surface, dialysate flow rate, and the puncture characteristics as distance, direction, gauge of the needle, and blood flow rate.^20^

Dias et al. reported that a distance between the needles greater than 5 cm is associated with lower blood recirculation in the vascular access and greater dialysis dose.^21^ Likewise, in our study, when the distance between tip of needles was greater than 5 cm, Kt/V was significantly higher. However, to reach this distance, a larger proportion of needles were inserted in retrograde position using 15G needles, suggesting that surface for cannulation was higher and the vascular access more developed. In combination, these characteristics allowed for greater blood flow and higher dialysis dose.

In summary, our study showed that when the distance between puncture needles is less than 5 cm, Kt/V was less than 1.4 in 59.6% of patients. When this distance is greater than 5 cm this proportion increased to 72.6%; however, this difference was not statistically significant. Therefore, even when the distance between the cannulas is less than 5 cm, it is still possible to reach the target dialysis dose if the other parameters of the prescription are properly adjusted.

Studies in which the distance between puncture needles was analyzed do not describe how distance was measured.^17^^,^ 21 In our study, the vascular access with the shortest distance between the needles was AVF (93%), proximal (55%), and with anterograde cannulation (98%). These characteristics suggest that fistulas probably had smaller extension for cannulation. Although anterograde cannulation is most commonly used in our center, the distance between the tip of needles was enough to allow a dialysis dose satisfactory for most patients. Therefore, retrograde cannulation is not a condition for an adequate dose of dialysis.

The needle bevel facilitates cannulation of the fistula. To prevent bleeding and pseudoaneurysms, it is very important that the bevel perforates the skin and the vein wall or the prosthesis without damaging these structures. The bevel usually faces up to favor the cutting angle; however, some cannulators prefer to introduce the needle with the bevel down in an attempt to reduce pain and diminish the risk of transfixing the posterior wall of the vascular access.^22^

Needles should have a back eye. This device favors the supply of blood to the dialysis circuit, as well as the return of blood to the systemic circulation.^12^ There is considerable controversy regarding needle rotation. While some authors contraindicate this technique because of the risk of endothelial trauma and a greater tendency for local bleeding^11^, others believe that rotating the needle after reaching the lumen of the vessel decreases the risk of accidental perforation of the posterior wall of the vascular access.^12^

In our dialysis unit, 100% of the needles have back eye, the puncture is performed with the bevel up, and the needles are always rotated in arterial punctures but not in venous punctures. These are the preferences of our unit and there is no study proving that our technique is superior to others. In relation to these variables, Gauly et al. reported that there is great variation among dialysis units, possibly because the local routine influence the technique.^17^

Regarding the diameter of the needles, 17G needles are the most used for new fistulas. When the AVF is mature, 14, 15, or 16G needles can be used, being the choice guided by the blood flow in the extracorporeal circuit. The higher the blood flow rate, the greater the caliber. Pressure control before the blood pump and venous pressure after the dialysis filter helps to choose the needle gauge.^12^^,^ 23 ^,^ 24

A blood flow rate inside the fistula between 800 and 1200 mL/min allows a blood flow rate on the extracorporeal circuit of 300 to 450 mL/min. Under these conditions, if the pressure in the arterial line is lower than 200 to 250 mmHg and in the venous line higher than 200 to 250 mmHg, it is possible that the caliber of the cannula needs review.^25^^,^ 26 In our study, we did not assess pressures in the arterial and venous lines.

Our study has several limitations. It is an observational study involving a small number of patients and performed in a single dialysis center. We did not evaluate the length and depth of vascular access, variables that may influence the choice of cannulation technique. However, we believe that our results are relevant because they provide additional information on a topic little discussed in the literature.

In 2011, guidelines on vascular access for hemodialysis from the UK Renal Association^13^ suggested that preserving the fistula through good puncture technique, particularly with regard to the prevention of aneurysms, is critical. These guidelines recommend the rope ladder and buttonhole techniques for cannulation of AVF and the rope ladder for AVG.^13^

The K-DOQI initiative on vascular access^12^ published in 2006 states that for AVF the puncture angle should be 25°, done with the needle bevel up, using a cannula with back eye, without needle rotation. The arterial puncture may be in the anterograde or retrograde direction and the venous puncture always in the anterograde direction. Needles must be withdrawn at the end of dialysis at the same angle used for the puncture, and pressure is applied to the vessel only after complete removal of the needles. With regard to AVG puncture, K-DOQI recommends that the angle of puncture be 45° without needle rotation in the superficial graft and with the needle rotation after reaching the lumen of the vessel in the deep graft in an attempt to reduce risk of transposing the posterior wall of the prosthesis.^12^

In summary, during vascular access cannulation, the following recommendations should be followed: locate the anterior puncture; choose the rope ladder technique; select a vein segment with enough length for cannulation; maintain 3 to 4 cm distance from the arteriovenous anastomosis; maintain 5 cm or more between arterial and venous punctures, and avoid cannulation at aneurysm or pseudoaneurysm sites.

**Figure 1:**
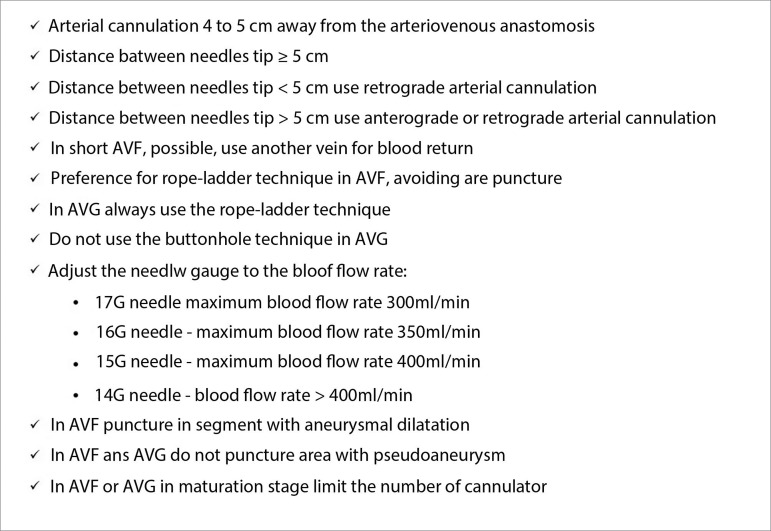
Recommendations for cannulation of arteriovenous fistula and arteriovenous graft in hemodialysis.

In our dialysis unit, we developed a continuing education program to standardize vascular cannulation technique. Figure 1 shows the recommendations being followed in our unit. Constant supervision reinforces the implementation of these standards. We believe that over time and with the trust of the team, it will be possible to modify beliefs and customs.

Finally, the ideal technique for vascular access cannulation is not fully established. The comparison of the results presented by different dialysis centers shows that the cannulation technique is very variable and subject to factors such as experience and local preference. Undoubtedly, the type of vascular access cannulation depends on the experience and training of cannulators and vascular access characteristics. Therefore, in the absence of randomized and controlled clinical trials comparing different cannulation techniques, each dialysis center should compare its results with those available in the literature. From this comparison, changes in vascular access cannulation technique can be done with the aim of reducing failure rates and improving arteriovenous fistula and graft survival.
